# Biogeography and phylogenetic position of *Enchodeloides signyensis* (Loof, 1975), gen. n., comb. n. from Maritime Antarctic (Nematoda, Nordiidae)

**DOI:** 10.3897/zookeys.697.13770

**Published:** 2017-09-14

**Authors:** Milka Elshishka, Stela Lazarova, Georgi Radoslavov, Peter Hristov, Vlada K. Peneva

**Affiliations:** 1 Institute of Biodiversity and Ecosystem Research (IBER), Bulgarian Academy of Sciences, 2 Gagarin Street, 1113 Sofia, Bulgaria

**Keywords:** Distribution, morphology, SEM, SNPs, taxonomy, 18S and D2-D3 rDNA

## Abstract

The taxonomic position of the endemic Antarctic species *Enchodeloides signyensis* (Loof, 1975), **gen. n.**, **comb. n.** (= *Enchodelus signyensis* Loof, 1975) is discussed on the basis of morphological study, including SEM, morphometric data, postembryonic observations, and sequence data of 18S rDNA and the D2-D3 expansion fragments of the large subunit rDNA. A number of characters such as the cuticle and stoma structures, including the presence of moderately developed cuticularised ring around the oral aperture, peculiarities of pharynx expansion, size and position of the posterior pair of pharyngeal nuclei, a less complex uterus, and the position of a posterior ventromedian supplement show that this species differs substantially from the other members of the genus *Enchodelus*. Furthermore, both the 18S and 28S rDNA-based phylogenetic trees of the *Enchodelus* sequences available in the GenBank formed two distinct clusters with *E. signyensis* being a part of a well-supported group with species of the genus *Pungentus*; therefore, it is proposed that its taxonomic position should be reconsidered.

## Introduction

Antarctic represents unique types of habitats – polar deserts, caused by its geological history, harsh climate conditions, and remoteness. Therefore, terrestrial Antarctic biota, including nematodes, is characterised by a very high degree of endemism and low diversity (Nielsen et al. 2011). Besides, distribution of nematodes exhibits clear biogeographical patterns regarding the two Antarctic ecozones, Continental and Maritime Antarctic (Andrássy 1998a). Order Dorylaimida Pearse, 1942 is represented in Antarctic by nineteen species (12 species described from Maritime Antarctic, 7 species, from the continental part, all but one endemics; of six genera reported from this polar region, two are endemic (Elshishka et al. 2015a). *Enchodelus signyensis* Loof, 1975 is the only representative of the genus *Enchodelus* Thorne, 1939 reported from the southern hemisphere and is an endemic for the Maritime Antarctic. This species was recorded from Signy Island (Spaull 1973) as *Enchodelus* sp. Later Loof (1975) studied Spaull’s collections from some of the islands and described this species as *E. signyensis*, naming it after the type locality. Subsequently, Andrássy (1998a) presented a brief description based on a female paratype specimen. Peneva et al. (2002) provided new morphological data about this species from Livingston Island, and described the males. Here new molecular and additional morphological data is presented of adults and juveniles of this species from Livingston and King George Islands, and its taxonomic position discussed.

## Materials and methods

Samples were collected from Livingston Island by Dr. N. Chipev (IBER), Dr. R. Mecheva (IBER), D. Apostolova (Sofia University) and from King George Island by Dr. R. Zidarova (Sofia University) during the regular Bulgarian Antarctic Expeditions (2006-2016). Nematodes were extracted from soils and plant materials by a Baerman funnel method (van Bezooijen 2006) for at least 48 hours, killed by gentle heat, and fixed in 4% formalin. For light-microscopy, specimens were processed in anhydrous glycerine (Seinhorst 1959) and mounted on permanent slides. Drawings were prepared using an Olympus BX 51 compound microscope, equipped with a drawing tube. Photographs were taken using an Axio Imager.M2-Carl Zeiss compound microscope equipped with a digital camera (ProgRes C7) and specialised software (CapturePro Software 2.8). Measurements were made using an Olympus BX 41 light microscope with a drawing tube and digitising tablet (CalComp Drawing Board III, GTCO CalCom Peripherals, Scottsdale, AZ, USA) and Digitrak 1.0f computer program (Philip Smith, John Hutton Institute, Dundee, UK).

Specimens used for SEM observations were rinsed in 0.1 M cacodylate buffer (twice for 10 min), post-fixed in 1% OsO_4_ for 2 h, washed twice for 10 min in 0.1 M cacodylate buffer and dehydrated in an ethanol series (Mutafchiev et al. 2013), immersed in hexamethyldisilazane for 30 min and air dried. They were sputter coated with gold in a JEOL JFS 1200 and examined using a JEOL JSM 5510 microscope at 10 kV.

The locations of pharyngeal gland nuclei are given following Loof and Coomans (1970) and Andrássy (1998b).

### DNA extraction, amplification, and sequencing

Genomic DNA was extracted from two female specimens per species using a standard nematode digestion protocol (Holterman et al. 2006). The specimens used for DNA extraction, amplification, and sequencing were from King George island (*E. signyensis*) and from Rila Mountain (*Enchodelus* sp.). For further details on the procedures used for DNA extraction, amplification, and sequencing, see Nedelchev et al. (2014). Identical sequences were obtained from both individuals of the same species and have been deposited in GenBank with the following accession numbers: for the 18S rDNA KY 881720 (*E. signyensis* gen. n., comb. n.) and KY766261 (*Enchodelus* sp.) and for D2-D3 rDNA KY881719 (*E. signyensis* gen. n., comb. n.) and KY766260 (*Enchodelus* sp.).

### Sequences and phylogenetic analyses

The 18S and D2-D3 28S rDNA sequences were compared with those of other nematode species available at the GenBank sequence database using BLASTN similarity search tool. The sequences revealing the highest similarity were used for sequence and phylogenetic analyses (Meldal et al. 2007; Holterman et al. 2008; Pedram et al. 2009, 2011a; Pedram et al. 2011b; Pedram et al. 2015, etc.). Bayesian Inference (BI) algorithm implemented in MrBayes 3.2.5 was used for reconstruction of phylogenetic relationships (Huelsenbeck and Ronquist 2001; Ronquist et al. 2012). For further details on phylogeny analyses and tree visualisation, see Lazarova et al. (2016). Based on previous studies (Holterman et al. 2006; Elshishka et al. 2015a) *Aporcelaimellus* spp. were selected as an outgroup for both phylogenies. The estimates of evolutionary divergences between sequences/species within and between groups (numbers of base differences and p-distances) were performed with MEGA7 (Kumar et al. 2016). The analyses involved nine nucleotide sequences with 790 and 1666 positions in total for D2-D3 and 18S rDNA, respectively.

## Taxon treatment

### Enchodelus signyensis

Taxon classificationAnimaliaDorylaimidaNordiidae

Loof, 1975

**Figure 1.**
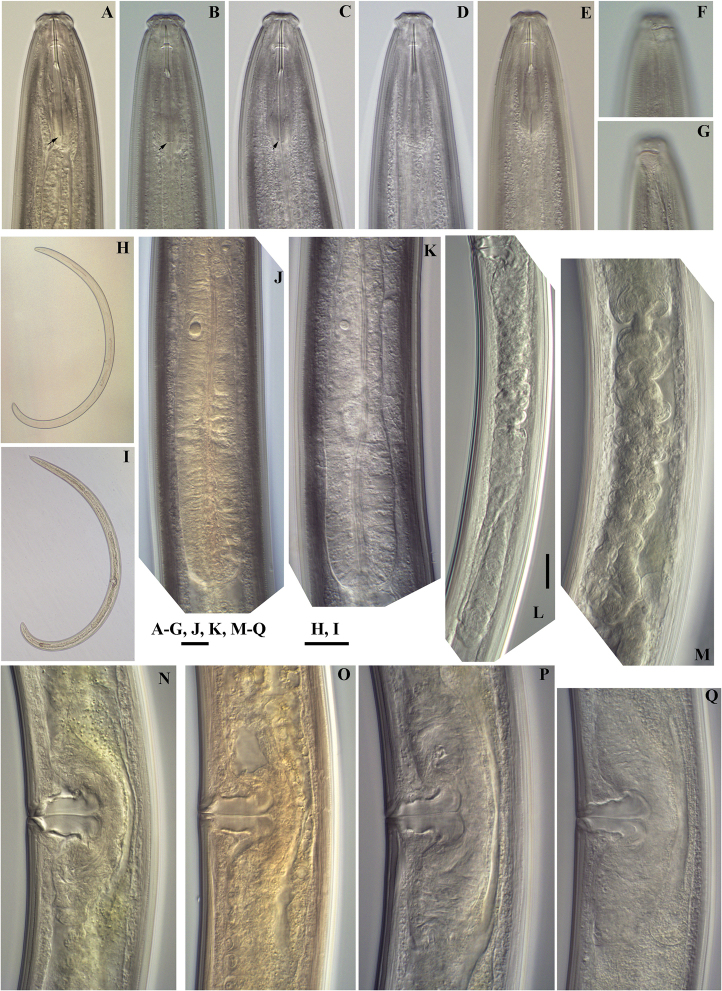
*Enchodeloides signyensis* (Loof, 1975), gen. n., comb. n. (= *Enchodelus signyensis* Loof, 1975). *Female*: **A–E** Anterior region (**A, B** specimens from Livingston Island **C, D, E** specimens from King George Island), black arrows indicate the minute basal swellings **F, G** Amphideal fovea (**E** specimen from Livingston Island **G** specimen from King George Island) **H, I** Entire body **J, K** Pharyngeal bulb (**J** specimen from Livingston Island **K** specimen from King George Island) **L** Posterior genital branch (specimen from Livingston Island) **M** Uterus (specimen from Livingston Island) **N–Q** Vulval regions (**N, O** specimens from Livingston Island **P, Q** specimens from King George Island). Scale bars: 10 μm (**A–G, J, K, M–Q**); 200 μm (**H, I**); 20 μm (**L**).

**Figure 2.**
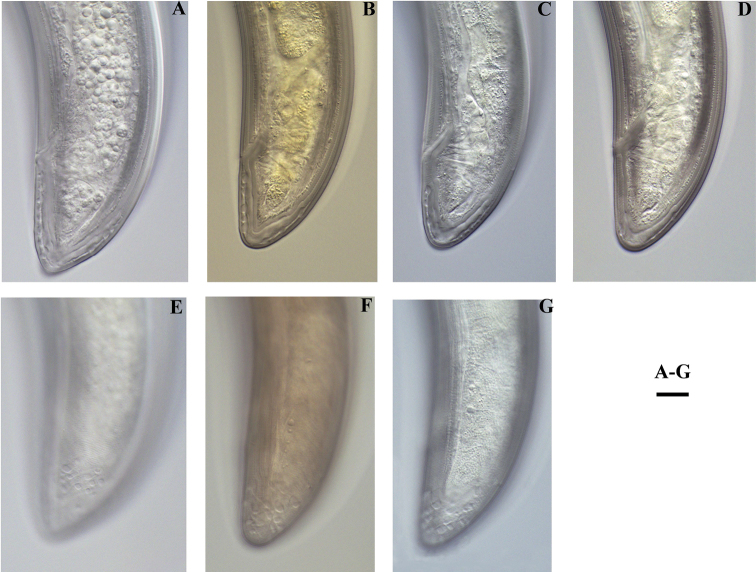
*Enchodeloides signyensis* (Loof, 1975), gen. n., comb. n. (= *Enchodelus signyensis* Loof, 1975). *Female*: **A–D** Tail ends (**A** specimen from King George Island; **B, C, D** specimens from Livingston Island) **E–G** Tail ends with saccate bodies (**E** specimen from King George Island **F, G** specimens from Livingston Island). Scale bar: 10 μm.

**Figure 3.**
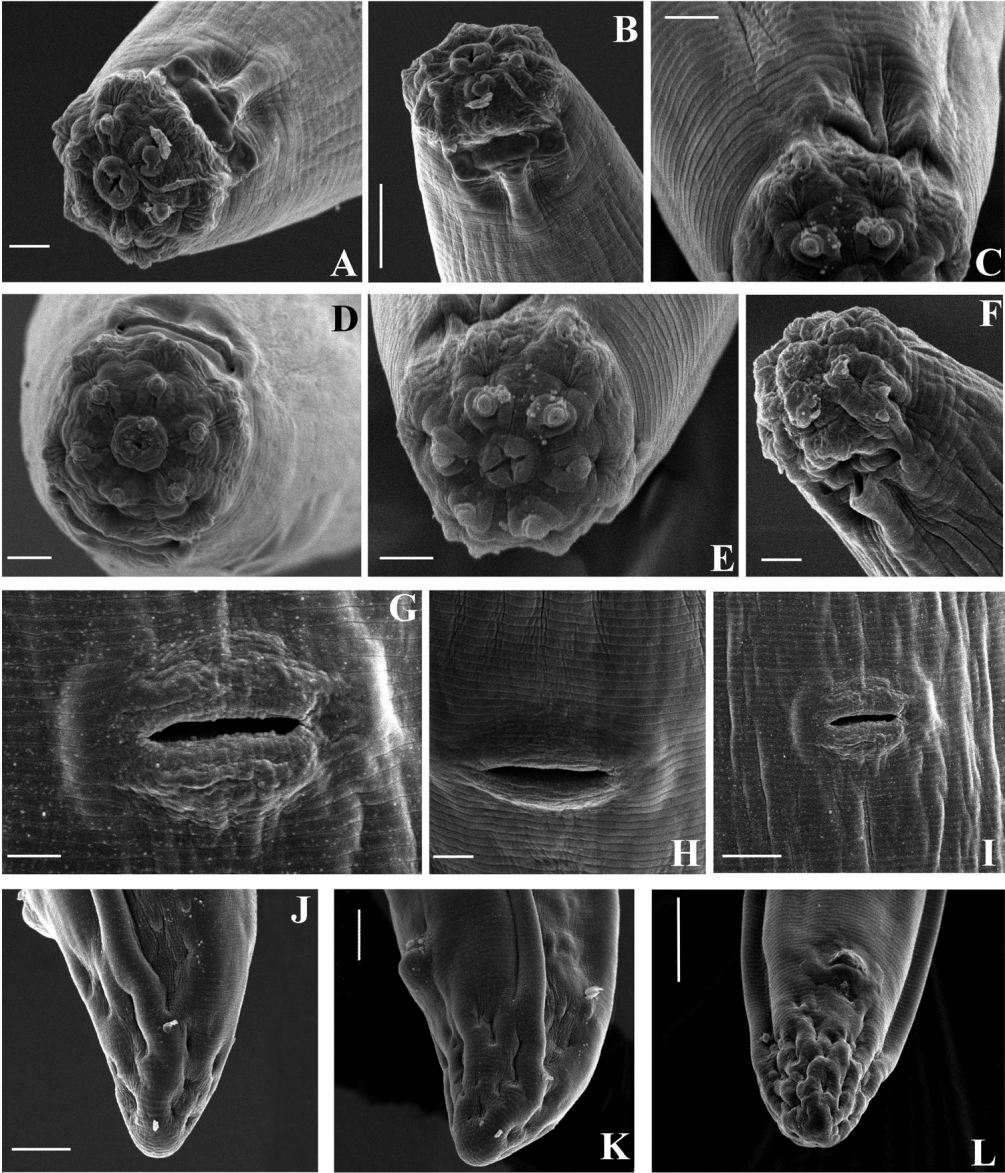
SEM micrographs. *Enchodeloides signyensis* (Loof, 1975), gen. n., comb. n. (= *Enchodelus signyensis* Loof, 1975). *Female*: **A, D, E** Lip region, in face view, amphid aperture **B, F** Lip region, in sublateral view **C** Cephalic and labial papillae **G–I** Vulval region **J–L** Tail ends. Scale bars: 2 μm (**A, C, D, E, F, G**); 5 μm (**B, I, J**); 10 μm (**L**).

**Figure 4.**
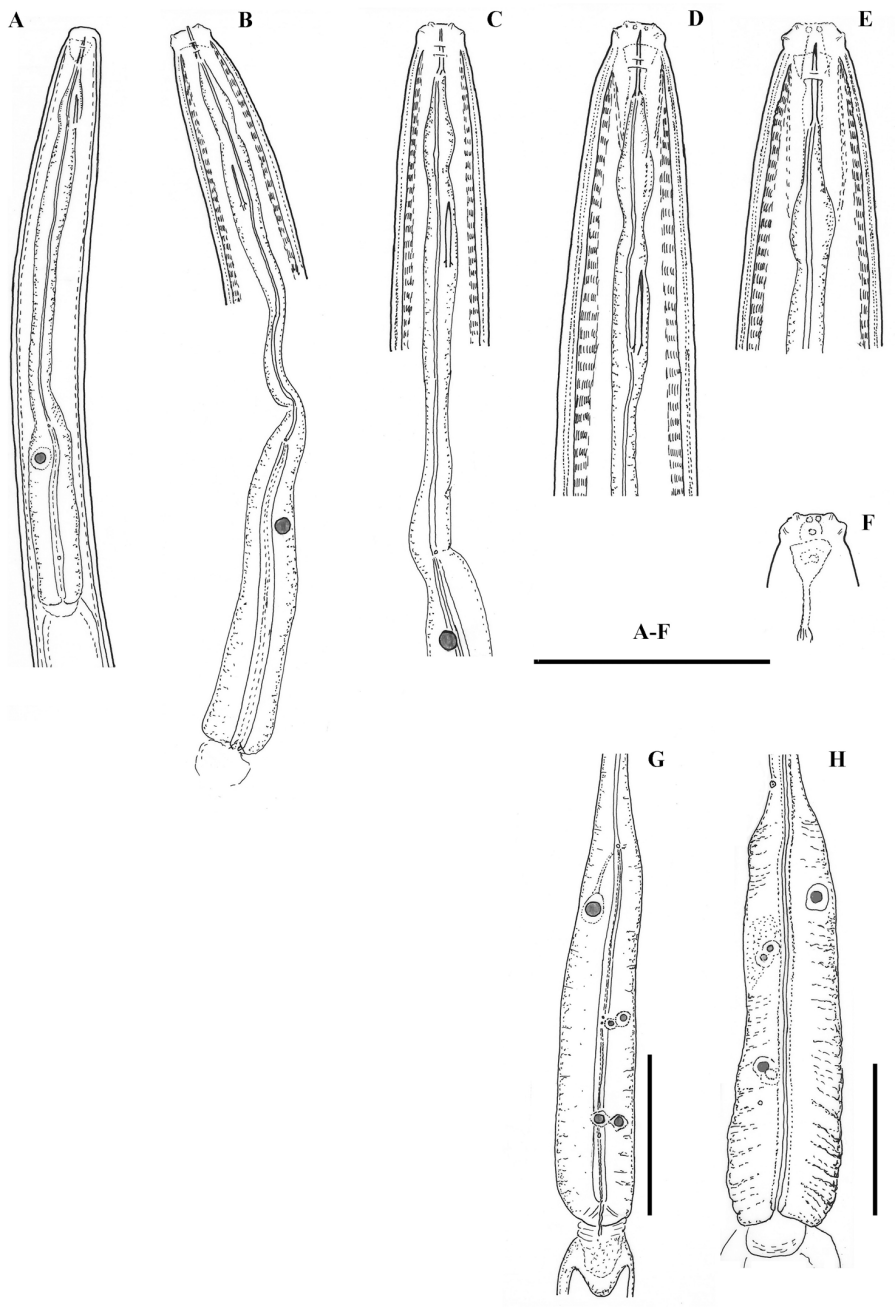
*Enchodeloides signyensis* (Loof, 1975), gen. n., comb. n. (= *Enchodelus signyensis* Loof, 1975). *Juveniles*: **A–D** Anterior ends (J1-J4) (specimens from Livingston Island) *Female* (specimen from Livingston Island) **E** Anterior end **F** Amphideal fovea **G** Pharyngeal bulb. *Enchodelus groenlandicus* (Ditlevsen, 1927) **H** Pharyngeal bulb. Scale bar: 50 μm.

**Figure 5.**
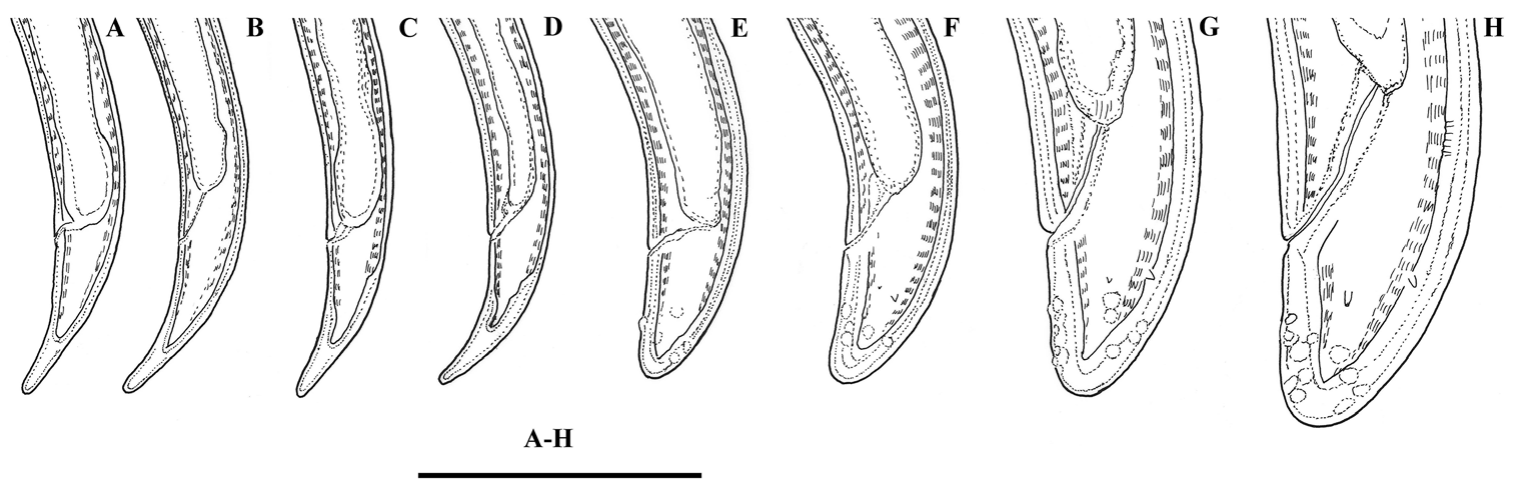
*Enchodeloides signyensis* (Loof, 1975), gen. n., comb. n. (= *Enchodelus signyensis* Loof, 1975). *Juveniles* (specimens from Livingston Island): **A–D** Tail ends (J1) **E–G** Tail ends (J2-J4) *Female* (specimen from Livingston Island) **H** Tail end. Scale bar: 50 μm.

**Figure 6.**
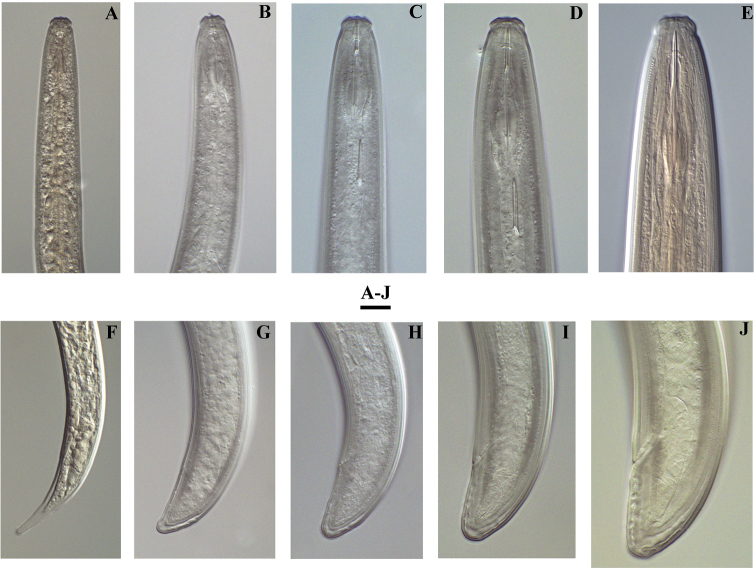
*Enchodeloides signyensis* (Loof, 1975), gen. n., comb. n. (= *Enchodelus signyensis* Loof, 1975). *Juveniles* (specimens from Livingston Island): **A–D** Anterior ends (J1-J4) **F–I** Tail ends (J1-J4) *Female* (specimen from Livingston Island) **E** Anterior end **J** Tail end. Scale bar: 10 μm.

Figs 1 , 2 , 3 , 4 , 5 , 6

#### Material examined.

Twenty-eight females and twenty-one juveniles (J1-J4) from Livingston and King George Islands (Table 1).

**Table 1.** Origin of the examined materials of *Enchodeloides signyensis* gen. n., comb. n.

Site description	Collection year	Abbreviation
**King George Island (KGI)**	–	–
***Fildes Peninsula*** /*Moist brown soil without vegetation, surrounded by moss*	2013	** KGI_F **
**Livingston Island (LI)**	–	–
***Svetilishteto***	2006–2007	** LI_SV **
***Playa Bulgara*** /*Mosses*	2008	** LI_M **
***Punta Hesperides*** /*Soil under moss crust*	2010	** LI_PH **
***Punta Hesperides*** /*Soil*	2016	** LI_PH_n **

#### Description.

Measurements. See Table 2–4.

**Table 2.** Morphometrics of *Enchodeloides signyensis* gen. n., comb. n. (females). All measurements, unless indicated otherwise, are in µm (and in the form: mean±SD (range)).

Locality	King George Island	Livingston Island			
Characters	**KGI_F**	**LI_SV**	**LI_PH**	**LI_M**	**LI_PH_n**
n	7	4	3	12	2
L (mm)	1.59±0.1 (1.47–1.66)	1.45±0.05 (1.39–1.49)	1.43; 1.51; 1.44	1.35±0.1 (1.20–1.45)	1.27, 1.37
a	28.9±1.9 (26.9–32.8)	28.1±1.2 (26.7–29.5)	28.5; 31; 27	29.5±1.4 (27.6–32.4)	26.1, 28.2
b	5.3± 0.3 (4.7–5.6)	4.8± 0.2 (4.6–4.9)	5; 5; 4.8	4.5± 0.2 (4.2–4.8)	4.1, 4.6
c	43.7±2.1 (40.6–46.9)	48.2±3.1 (43.8–50.7)	49.3; 48; 44.8	43.7±3.9 (37–50)	50.1, 53.2
c‘	1.0±0.04 (1.0–1.1)	1.0±0.1 (0.9–1.0)	0.9; 1.0; 1.0	1.0±0.1 (0.8–1.1)	0.9, 0.9
V %	50.4±0.7 (49.5–51.5)	53.8±1.3 (52–55)	51; 54; 53	54.4±1.0 (52–56)	55, 56
Lip region diameter	14.3±0.4 (14–15)	14.2±0.2 (14–14.4)	14; 14; 15	14.1±0.7 (13–15)	14, 13
Odontostyle length	19.9±0.8 (19–21)	18.9±0.7 (18–19.5)	19; 20; 20	19.2±0.8 (18–20)	18, 19
Odontophore length	25.2±0.8 (24–26.5)	26.5±0.4 (26–27)	25; 23.5; 25	26.4±2.8 (22–32)	27, 26
Anterior end to guiding ring	12.0±0.7 (11–13)	12.6±0.3 (12–13)	12; 11; 12	12.1±0.6 (11–13)	12, 12
Pharynx length	297.8±11.4 (277–310)	304.0±3.8 (302–308)	283; 301; 297	302.2±11.8 (271–314.5)	307, 300
Pharyngeal base diameter	51.5± 3.8 (45–55)	46.7±2.4 (44–49)	47; 45; 47.5	43.3± 2.9 (38–46.5)	45, 45.5
Mid-body diameter	55.2±3.5 (50–60)	51.8±1.8 (49–54)	50; 49; 53	45.9±3.8 (39–51)	48, 48.5
Prerectum length	104.2±32.2 (72–166)	–	71	84.5±24.5 (62–128)	-, 75
Rectum length	36.4±3.0 (32–40)	32, 46	30.5; 37; 41	33.4±2.0 (31–36.5)	-, 37.5
Tail length	36.4±2.1 (32–39)	30.3±2.7 (28–34)	29; 31.5; 32	31.2±3.4 (25–35)	25, 26

*Female.* Habitus curved ventrally after fixation, adopting a C-shape. Cuticle consisting of four layers with different refraction, the outer two layers thinner, the second outer with stronger refraction, the inner layers thicker, especially at tail region. Cuticle 2–3 µm thick at postlabial region at the level of the guiding ring, 2–4 µm at mid-body and 4–6 µm on tail; outer layer with very fine transverse striations, innermost layer coarsely striated (Figs 1, 2). Lip region 4–5 μm high angular (following terminology adopted by Peña-Santiago (2006)), offset from the adjoining body by a constriction; about 3 times as wide as high. Based on SEM photographs (Fig. 3), perioral area high, disc-like structure with apparently four elevations surrounding oral aperture, oral aperture appearing cross-like in shape in frontal view. Labial and cephalic papillae prominent; labial papillae button-like, each surrounded by a small ring, their openings pore-like. Inner labial papillae located at distinct elevations; separated from each other, and far from oral aperture and outer labial papillae; divided from the outer labial and cephalic papillae by a circular striation (Fig. 3). Cephalic papillae button-like; outer labial and cephalic papillae below the margin of oral field. Six radial striations beginning from the oral field interrupted by inner and ending at outer labial papillae. Amphidial fovea cup-shaped, its aperture approximately half of lip region diameter, its margin curved; under SEM, the amphidial aperture with an operculum, however the presence of this structure should be confirmed with further studies. Cheilostom a truncate cone with weakly developed walls, its anteriormost part representing a moderately cuticularised perioral ring, appearing as small perioral refractive dots. Odontostyle short and slender, straight, 18–20 times as long as wide, 1.2–1.6 times lip region diameter, aperture 14–16% of its length, 1.2–1.7% of body length. Odontophore 1.2–1.6 times as long as odontostyle, with small swellings at its base. Guiding ring double, located at 0.8–1.0 times lip region diameter from anterior end. Anterior region of pharynx enlarging gradually; pharyngeal expansion 112.5–134 µm, occupying 37–45% of total pharynx length. Location of pharyngeal gland nuclei and their orifices is presented in Table 3. Distance DO-DN 14–19 μm, nuclei of dorsal and second ventrosublateral glands clearly visible, nuclei of first ventrosublateral glands in most specimens indistinct, located slightly behind the middle of the distance DN-S_2_N (n = 1). Nuclei of dorsal glands 3.5–5 μm diameter, first and second pair ventrosublateral 1 μm and 2–3 μm, respectively. Excretory pore opposite the nerve ring with slightly cuticularised canal clearly visible at 100–112 μm from the anterior end. Cardia rounded conoid. Prerectum 1.7–4.8, rectum 0.9–1.4 times anal body diameter long. Tail bluntly conoid, 2–3% of body length, with numerous saccate bodies. Hyaline part 4–8 μm wide or 12–25% of tail length. Two pairs of caudal pores present. Both branches of female genital system equally and well-developed (in specimens of Livingston Island shorter: anterior 236.2 ± 23.3 (186–275) µm and posterior 208.2 ± 34.4 (143–259) µm long, in specimens from King George Island anterior 298.3 ± 31.9 (245–330) µm and posterior 323.1 ± 46.4 (243–361) µm long). Ovaries short, rarely reaching sphincter level; oviduct with well-developed *pars dilatata*. Sphincter well developed. Uteri tubular, thick walled, surrounded by hyaline cells along almost the whole length, anterior uterus 104–152 µm long, posterior 105–156 µm long, 2–3 times corresponding body diameter, not differentiated. Vulva a transverse slit. Vagina extending inwards for 54–76% of body diameter; *pars proximalis* 19.5–25×12–15 μm, *pars refringens* with two drop shaped sclerotised pieces, with combined width of 11–13 μm, *pars distalis* 4–5 μm long.

**Table 3.** Morphometrics of *Enchodeloides signyensis* gen. n., comb. n. (juveniles). All measurements, unless indicated otherwise, are in µm (and in the form: mean±SD (range)).

Locality	Livingston Island					King George Island
Characters	** LI_S **	**LI_M**			**LI_PH_n**	**KGI**
Stages	J1	J2	J3	J4	J4	J4
n	6	1	1	7	1	4
L (mm)	0.40±0.1 (0.37–0.42)	0.60, 0.62	0,7	1.02±0.1 (0.93–1.13)	1.03	1.23±0.1 (1.07–1.34)
a	26.5±1.1 (24.7–27.9)	27.0, 27.5	27	28.9±1.8 (26.8–31.7)	26.9	28.3±1.6 (26.9–30.4)
b	3.2±0.5 (2.9–4.2)	3.6, 3.8	3,5	3.9± 0.2 (3.7–4.1)	–	4.9, 5.2
c	13.6±0.9 (12.8–14.8)	24.7, 26.2	27,6	36.8±2.3 (33.7–39.6)	35.5	39.0±2.7 (36.5–42.8)
c‘	2.8±0.2 (2.6–3.1)	1.5, 1.5	1,4	1.1±0.1 (1.0–1.2)	1.1	1.0±0.05 (1.0–1.1)
Lip region diameter	7.3±0.2 (7–7.5)	8.5, 8	11	11.8± 0.3 (11–12)	11	12.2±0.4 (12–12.5)
Odontostyle length	6.6±0.4 (6–7)	8, 8	11	14.7±0.2 (14–15)	15	15.6±0.2 (15–16)
Replacement odontostyle length	8.2±0.2 (8–8.3)	11, 10	14	18.5±0.4 (18–19)	20	19.3±0.9 (18–20)
Pharynx length	126.2±15.9 (95–140)	165.5, 163	200.5	258.8±14.2 (244–281)	–	207, 249
Pharyngeal base diameter	16.0±0.3 (15.6–16.3)	23, 23	27	35.3±2.5 (32–40)	36	40.5±3.1 (37–44)
Mid-body diameter	15.0±0.4 (14–15.5)	22, 23	26	35.4±2.9 (31–40)	38.5	43.4±4.3 (40–47)
Prerectum length	35	–	–	87, 110	–	76, 86, 82
Rectum length	14,5	–	–	25.5±2.7 (21.5–28.5)	26	33, 30, 35
Tail length	29.6±2.2 (27–31)	24, 24	25	27.7±1.2 (26–30)	29	31.4±2.1 (29–34)

**Table 4.** Pharyngeal characters of *Enchodeloides signyensis* gen. n., comb. n. For abbreviations see Loof & Coomans (1970) and Andrássy, 1998b.

	LI_	LI_PH	LI_S	KGI_F
DN=D	67–70	69	72, 68	63–67
DO	64, 64, 62	63	66, 62	55–63
S_1_N_1_	–	–	80	–
S_1_N_2_	–	–	79	–
S_2_N	89–91	90, 91	92, 90	89–90
S_2_O	92	–	93	90, 91
AS_1_	–	–	37	–
AS_2_	–	–	35	–
PS_1_	65–71	70	71, 68	67–74
PS_2_	66–72	68	70, 69	67–72

**Figure 7.**
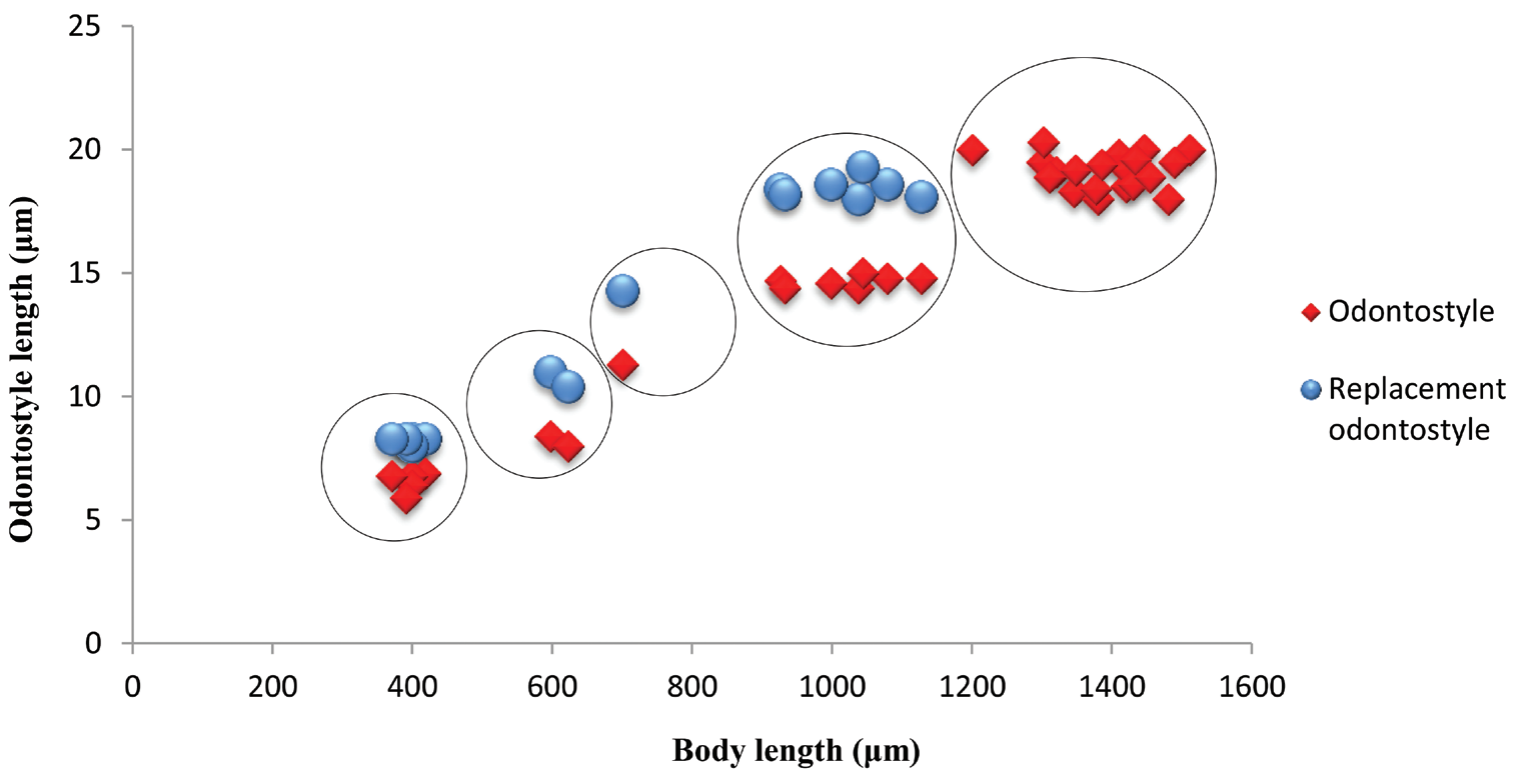
*Enchodeloides signyensis* (Loof, 1975), gen. n., comb. n. (= *Enchodelus signyensis* Loof, 1975). Scatter plot of the functional (○) and replacement odontostyle (◊) in relation to the body length of the juvenile stages and females.

*Juveniles.* Based on morphometrics of juvenile specimens and the relationships between the lengths of their functional and replacement odontostyles and body lengths, four juvenile stages were identified (Figs 4–7). Habitus in first juvenile stage slightly ventrally curved, lip region flat, continuous with the body, genital primordium 11–12 μm long, tail conical elongated with long central peg (Figs 4–6). Tail in J2 and J3 conoid elongated in J4 bluntly conoid as in females with numerous saccate bodies on tail, c’ decreasing during the successive stages to J4 and females.

**Figure 8.**
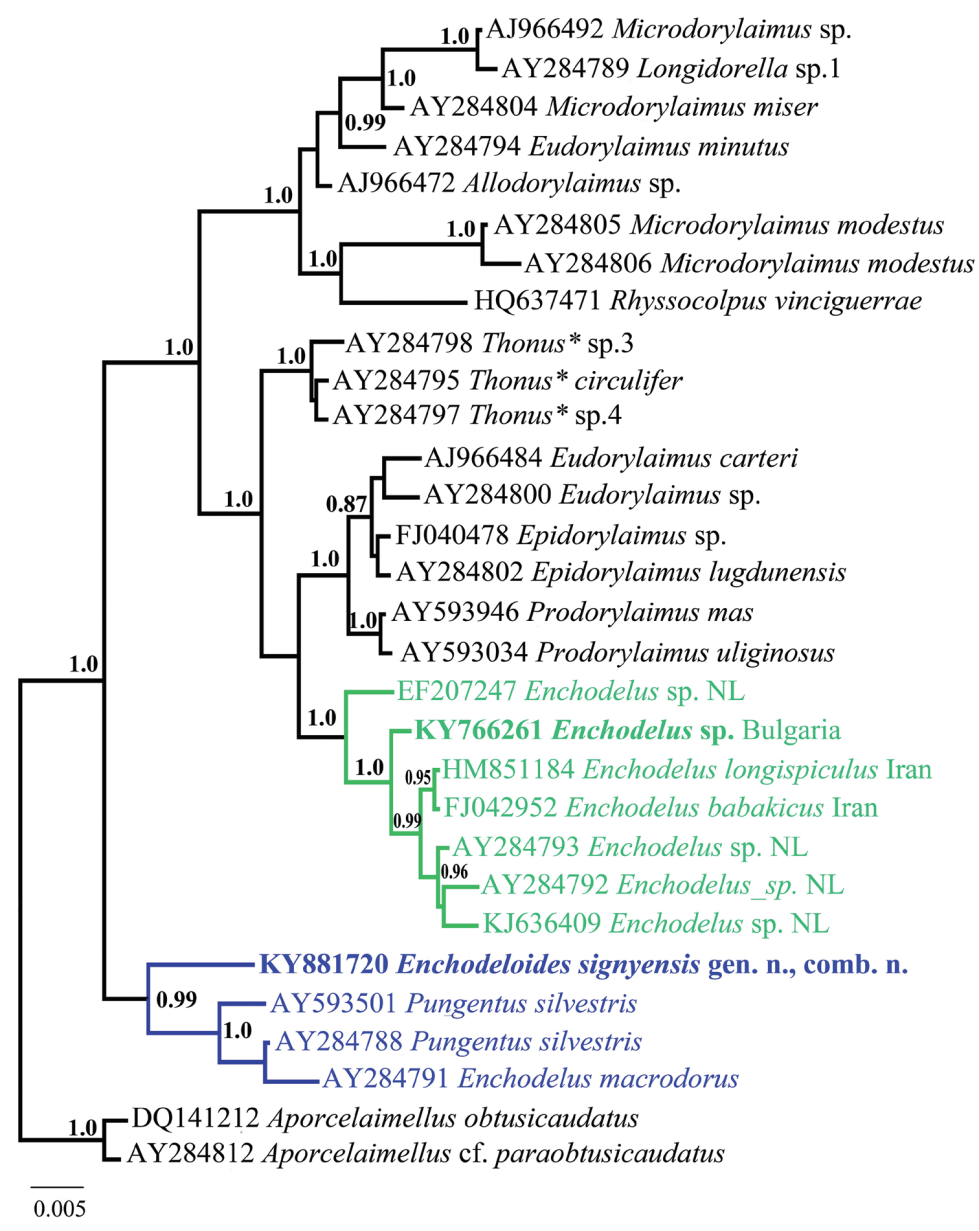
Phylogenetic relationships of *Enchodeloides signyensis* (Loof, 1975), gen. n., comb. n. (= *Enchodelus signyensis* Loof, 1975) based on 18S rDNA inferred from a Bayesian analysis (GTR+G model) and two *Aporcelaimellus* species used as an outgroup. * *Thonus* is currently considered a synonym of *Crassolabium* (Peña-Santiago and Ciobanu, 2008).

**Figure 9.**
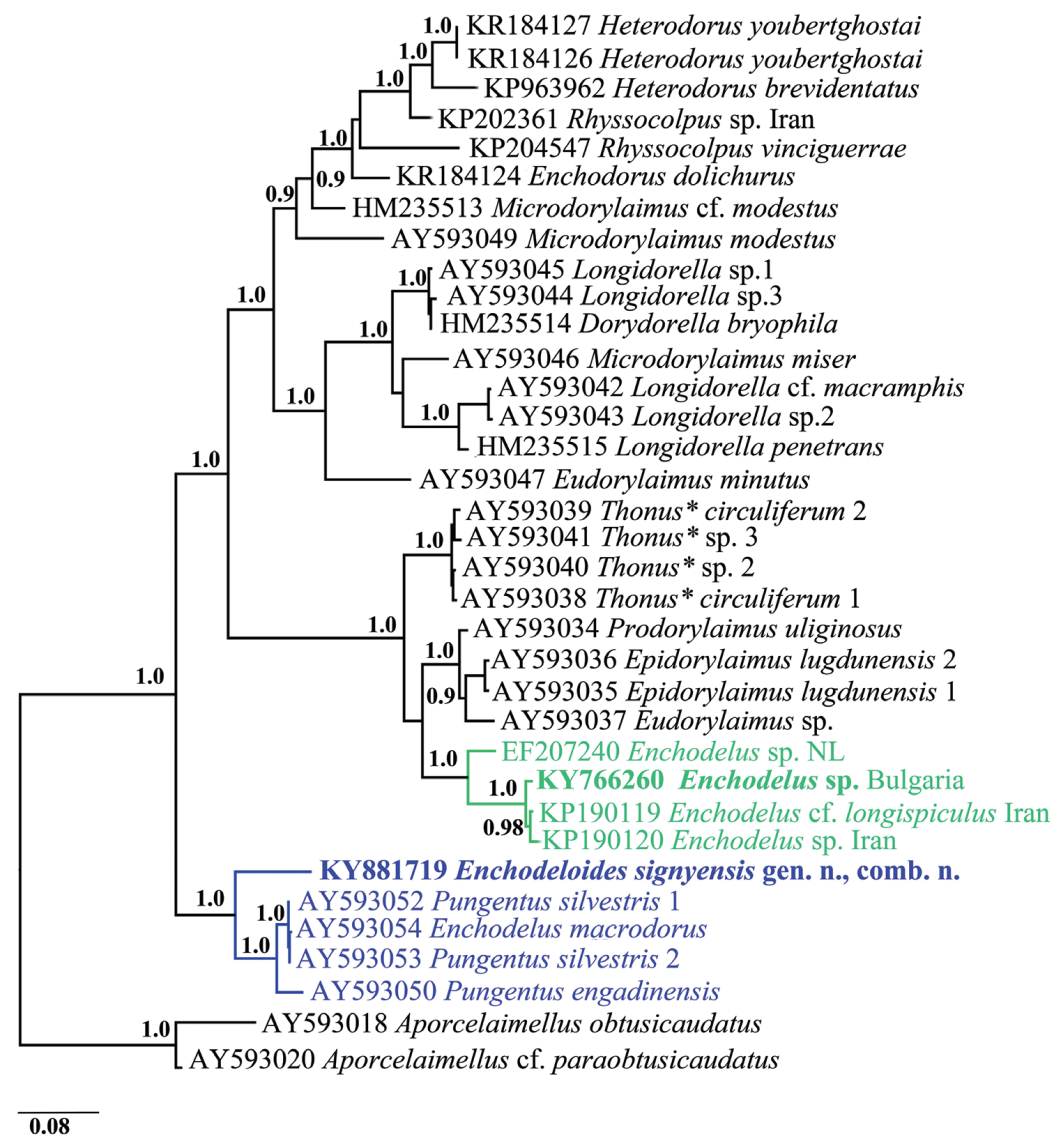
Phylogenetic relationships of *Enchodeloides signyensis* (Loof, 1975), gen. n., comb. n. (= *Enchodelus signyensis* Loof, 1975) based on 28S rDNA D2-D3 inferred from a Bayesian analysis (GTR+G model) and two *Aporcelaimellus* species used as an outgroup. * *Thonus* is currently considered a synonym of *Crassolabium* (Peña-Santiago & Ciobanu, 2008).

*Sequences and phylogenetic analyses.* The phylogenies based on both gene regions showed that *Enchodelus* sp. and *E. signyensis* are parts of two distantly related and well-supported groups (I and II), and in both analyses, they revealed similar relationships with other dorylaimid species (Figs 8, 9). With one exception (AY593052, *E. macrodorus* (de Man, 1880) from The Netherlands), *E. signyensis*, was evolutionary close to *Pungentus* spp. (AY593050, AY593052–53 for D2-D3 28S, and AJ966501 and AY284788 for 18S rDNA) while, *Enchodelus* sp. from Bulgaria clustered with other *Enchodelus* spp. from the Netherlands and Iran being a part of well-supported clade including species of various genera (*Eudorylaimus* Andrássy, 1959, *Epidorylaimus* Andrássy, 1986, *Prodorylaimus* Andrássy, 1959 and *Crassolabium* Yeates, 1967).

The estimates of evolutionary divergences (p-distances) between D2-D3 28S rDNA sequences within and between both groups are presented in Table 5. The dissimilarity between *E. signyensis* and other *Enchodelus* spp. is very high, varying from 16.6% to 17.1% while within **group II** the distances between sequences are between 0.8–7.1%. The dissimilarity within **group I** varies from 0.1% to 7.6% with the highest values (7.4–7.6%) estimated from pair-wise comparison of *E. signyensis* to other sequences within the group. A similar pattern was observed when 18S rDNA evolutionary divergences were analysed. Although having much lower resolution, the 18S rDNA distance of *E. signyensis* to other *Enchodelus* species available at NCBI was 2.6–2.8% (or 44–47 nucleotides). This species was the most closely related to two *Pungentus* spp. from Europe (AJ966501 and AY284788) showing 1.4–1.6% dissimilarity (or 24–26 nucleotides difference). The SNPs analyses of the parsimony-informative sites between sequences for *Enchodeloides* gen. n., *Enchodelus* and *Pungentus* Thorne & Swanger, 1936 and for both genes are given as Suppl. materials 1 and 2.

**Table 5.** Genetic distances using D2-D3 28S rDNA sequence data (p-distances given in percents). Pair-wise comparisons are based on alignment with 790 nucleotide positions (all positions containing gaps were eliminated).

	Sequence number/species	1	2	3	4	5	6	7	8	9
**1**	**KY881719*E. signyensis* gen. n., comb. n., Antarctica**									
**2**	AY593050 *Pungentus engadinensis* (Altherr, 1950) Altherr, 1952	**7.6**								
**3**	AY593052 *Pungentus silvestris* (de Man, 1912) Coomans & Geraert, 1962, 1 NL	**7.4**	2.7							
**4**	AY593053 *P. silvestris* 2, NL	**7.5**	2.8	0.1						
**5**	AY593054 *Enchodelus macrodorus* (de Man, 1880) Thorne, 1939, NL	**7.5**	2.8	0.1	0.3					
**6**	KY766260 *Enchodelus* sp., Bulgaria	**17.1**	17.3	16.0	16.2	16.2				
**7**	EF207240 *Enchodelus* sp., NL	**16.6**	16.1	14.9	15.0	15.0	6.5			
**8**	KP190119 *E. longispiculus* Guerrero, Liébanas & Peña-Santiago, 2008, Iran	**17.0**	17.1	15.9	16.0	16.0	0.8	6.3		
**9**	KP190120 *Enchodelus* sp. 1, Iran	**17.1**	17.3	16.0	16.2	16.2	1.5	7.1	1.2	

#### Discussion.

Based on the main morphological characters, the studied populations are very similar, but specimens from King George Island differ by a somewhat longer (average 1.47–1.66 *vs* 1.20–1.51 mm), and wider body (55.2 ± 3.5 (50–60) µm *vs* 48.0±3.9 (39–54) µm), longer female genital branches (anterior 298.3±31.9 (245–330) µm and posterior 323.1 ± 46.4 (243–361) µm *vs* 236.2 ± 23.3 (186–275) µm and 208.2 ± 34.4 (143–259) µm, respectively, vulva position (V=50.4 ± 0.7 (49.5–51.5)% *vs* V=54.1±1.3 (51–56)%), and tail (32–39 *vs* 25–35 µm). The specimens examined generally agree well with data previously reported for this species (Loof 1975; Andrássy 1998a; Peneva et al. 2002), although some minor differences occurred: our populations have somewhat shorter body length (1.20–1.66 *vs* 1.37–1.88 mm) and the presence of a moderately developed cuticularised ring around the oral aperture has not been described in those studies. Although *E. signyensis* resembles members of the genus *Enchodelus* in many respects, this structure has not been reported for any of its species. The number of morphological characters (see below), as well as molecular data, do not support the current taxonomic position of this species as a member of the genus *Enchodelus* and therefore a new genus *Enchodeloides* gen. n. is proposed.

### Enchodeloides gen. n.

Taxon classificationAnimaliaDorylaimidaNordiidae

http://zoobank.org/0AFC0BD5-CA16-4A19-9165-16CD7EE71176

#### Diagnosis.

Nordiidae. Nematodes of medium size. Cuticle dorylaimoid, consisting of four layers, outer layer finely, inner layer coarsely transversally striated. Lip region angular; stoma entrance surrounded by a moderately developed cuticularised ring, appearing as small perioral refractive dots. Amphidial fovea cup-shaped, its aperture about half of lip region diameter, curved. Odontostyle short and slender, straight. Odontophore with small swellings. Guiding ring double. Anterior region of pharynx enlarging gradually into pharyngeal expansion. Posterior pair of pharyngeal nuclei smaller than dorsal nucleus, located posteriorly in pharyngeal expansion. Cardia rounded conoid. Female genital system amphidelphic. Uterus not differentiated. Vagina moderately sclerotised. Vulva a transverse slit. Males rare. Spicula stout ventrally curved. Lateral guiding pieces present. Sperm cells spindle-shaped. Supplements 2 to 4 in number preceded by an ad-cloacal pair of papillae, starting far behind the level of the spicules. Tail bluntly conoid, with numerous saccate bodies on tail. First juvenile stage with elongate conical tail with long central peg.

#### Relationships.

The new genus resembles members of the subfamily Pungentinae Siddiqi, 1969, especially the genera *Enchodelus*, *Pungentella* Andrássy, 2009, *Pungentus* and *Stenodorylaimus* Álvarez-Ortega & Peña-Santiago, 2011. It differs from *Enchodelus* by having lip region with six radial striae starting from inner and ending at outer labial papillae *vs* absent (seen under SEM), four *vs* three layered cuticle, two *vs* one thicker inner layer at tail region (under light microscopy), cheilostom thin walled *vs* thick walled, a moderately developed cuticularised ring around the oral aperture *vs* absent; less developed *vs* well developed basal swellings; a pharynx enlargement gradually expanding *vs* abruptly expanding into basal expansion (Fig. 4G, H), the posterior pair of pharyngeal nuclei generally smaller than dorsal nucleus *vs* as large as dorsal nucleus (Andrássy 2009), except for *E. macrodorus* Thorne, 1939 (Guerrero and Peña-Santiago 2007) and located more posteriorly, more than 89% *vs* 83–88% of the pharyngeal expansion (Loof and Coomans 1970); less complex uterus *vs* tripartite (bipartite in *E. distinctus* Ahmad & Jairajpuri, 1980 and *E. ponorensis* Popovici, 1995); posteriormost ventromedian supplement located at a considerable distance from the adcloacal pair and outside of the spicule range *vs* posteriormost one or two ventromedian supplements rather close to the adcloacal pair and inside the spicule range, 2–4 *vs* 7–16 in number, and finally, all representatives of the genus *Enchodelus* have been reported only from the northern hemisphere. *Enchodeloides* gen. n. differs from *Pungentella* by having transversally striated cuticle *vs* smooth; a longer odontostyle (much longer *vs* equal to or slightly longer than lip region diam.) with a smaller aperture (up to one-sixth *vs* one-fourth to one-third its length); a moderately developed cuticularised ring *vs* four small platelets around the oral aperture and the guiding ring double *vs* simple. From *Pungentus* it differs in having a moderately developed cuticularised ring *vs* four distinct circumoral platelets around the oral aperture; a straight *vs* arcuate odontostyle; shorter odontostyle (1.2–1.6 times *vs* 2–3 times lip region diameter (Andrássy 2009a); the first pair of ventrosublateral pharyngeal gland nuclei indistinct, difficult to observe *vs* well developed; a long distance DO-DN (5–6% *vs* 2–4% (Loof and Coomans 1970)); ventromedian supplements located at a considerable distance from the adcloacal pair and outside of the spicule range *vs* posteriormost 1–4 supplements lying within the spicule range, and with *vs* without hiatus. From the genus *Stenodorylaimus* it differs by having a shorter body (L=1.2–1.9 *vs* 3.7–5.1 mm), and a slender *vs* more robust odontostyle (1.2–1.7 *vs* 0.51–0.87% of body length); a longer pharynx (b-ratio up to 6 *vs* more than 7); saccate bodies present *vs* absent; the first pair of ventrosublateral pharyngeal gland nuclei indistinct, difficult to observe *vs* well developed; ventromedian supplements spaced *vs* irregularly spaced, 2–4 *vs* 14–19 in number, and with *vs* without hiatus.

Consequently, the new combination *Enchodeloides signyensis* (Loof, 1975) is proposed to accommodate the only nordiid species occurring in Maritime Antarctic.

## Distribution

*Enchodeloides signyensis* is a widespread endemic for the Maritime Antarctic, occurring in several islands: Signy (Loof 1975; Maslen 1981; Caldwell 1981), Coronation, Elephant, Galindez, Blaiklock (Loof 1975), Alamode (Loof 1975; Maslen and Convey 2006), Dream (Shishida and Ohyama 1989), Charcot (Convey et al. 2000; Maslen and Convey 2006), Livingston (Peneva et al. 2002, 2004; Elshishka et al. 2015b), Alexander (Maslen and Convey 2006), and King George Islands (Russell et al. 2014). It has been recorded from various microhabitats, different moss and algae communities, and in association with species of higher plants, reported from Maritime Antarctic (*D. antarctica* and *C. quitensis*) (Table 6). Data from previous records and the present study show that *E. signyensis* is associated with different type of microhabitats. Like other terrestrial nematodes in extreme polar conditions, a majority of which colonise all microhabitats, this species does not show specific biotope preferences. According to Chernov et al. (2011) the major life strategy of organisms inhabiting extreme environments is the development of tolerance and plasticity, not specialisation and competitiveness, which is typical of other biomes.

**Table 6.** Distribution of *Enchodeloides signyensis* gen. n., comb. n. in Antarctic islands and habitats.

**Island**	**Microhabitats and plant associations**	**References**
Signy	*Tortula excelsa* Card (type host) *Deschampsia antarctica* Desv. *Colobanthus quitensis* (Kunth) Bartl.	Loof 1975
	*Polytrichastrum alpinum* (Hedwig), *Chorisodontium aciphyllum* (Hook. f. & Wilson) Broth., *Sanionia uncinata* (Hedw.), *Calliergon sarmentosum* (Wahlenb.), *Calliergidium austro-stramineum* (C. Muell.) Bartr.	Maslen 1981
	*P. alpinum*, *Ch. aciphyllum*, *S. uncinata*, *C. sarmentosum*, *Cephaloziella varians* (Gottsche) Steph., soils contaminated by vertebrate, e.g. close to seabird nests	Caldwell 1981
Coronation	*D. antarctica*	Loof 1975
Elephant	*D. antarctica* ****** *Polytrichum* sp.	
Galindez	*D. antarctica*	
Blaiklock	*P. alpinum, Pohlia nutans* (Hedw.)	
Alamode	*S. uncinata*	
	Moss	Maslen and Convey 2006
Dream	Moss mats with green algae	Shishida and Ohyama 1989
Charcot	Soil, moss clumps, algae, various lichens	Convey et al. 2000
	Moss, lichen and soil	Maslen and Convey 2006
Livingston	*D. antarctica*, *S. uncinata*, *Sanionia georgico-uncinata* (Müll. Hal.) Ochyra & Hedenäs, *C. quitensis*, *P. alpinum*, *Bryum* sp., *Usnea* sp., *Cladonia* sp., *Polytrichum juniperinum* Hedw., *Bartramia patens* Brid.	Peneva et al. 2002
	Moss; soil under moss crust; soil	Present study
Alexander	Moss; lichen; soil; microbial mat	Maslen and Convey 2006
King George	*D. antarctica*, *C. quitensis*, *Sanionia* sp., *Syntrichia filaris* (Müll.Hal.), *Syntrichia magellanica* (Mont.)	Russell et al. 2014
	Moist brown soil without vegetation, surrounded by moss	Present study

## Supplementary Material

XML Treatment for Enchodelus signyensis

XML Treatment for Enchodeloides

## Supplementary materials

Supplementary material 1 18S Parsimoni informative sites TemporaryMEGA17 Data type: (nucleotide) Explanation note: 18S rDNA Phylogenetic analysis between tree Genus based on Parsimony informative nucleotide sites. This dataset is made available under the Open Database License (http://opendatacommons.org/licenses/odbl/1.0/). The Open Database License (ODbL) is a license agreement intended to allow users to freely share, modify, and use this Dataset while maintaining this same freedom for others, provided that the original source and author(s) are credited.Milka Elshishka, Stela Lazarova, Georgi Radoslavov, Peter Hristov, Vlada K. Peneva

Supplementary material 2 28S Parsimoni informative sites TemporaryMEGA15 Data type: (nucleotide) Explanation note: 28S rDNA Phylogenetic analysis between tree Genus based on Parsimony informative nucleotide sites. This dataset is made available under the Open Database License (http://opendatacommons.org/licenses/odbl/1.0/). The Open Database License (ODbL) is a license agreement intended to allow users to freely share, modify, and use this Dataset while maintaining this same freedom for others, provided that the original source and author(s) are credited.Milka Elshishka, Stela Lazarova, Georgi Radoslavov, Peter Hristov, Vlada K. Peneva
